# Integrating Molecular Biology and Cryptography: A DNA and RNA-Based Framework for Secure Data Encryption

**DOI:** 10.3390/ijms27104522

**Published:** 2026-05-18

**Authors:** Muhammad Naeem Akhtar, Jawad Hussain Awan, Abdul Mateen Shahzaib Asad, Min Young Kim

**Affiliations:** 1 Faculty of Science and Technology, University of Sufism and Modern Sciences, Bhitshah 70140, Pakistan; 2 Faculty of Engineering, Sciences and Technology, Iqra University, Karachi 75500, Pakistan; 3 School of Electronics Engineering, Kyungpook National University, Daegu 41566, Republic of Korea

**Keywords:** DNA cryptography, RNA transcription encryption, molecular computing, bio-inspired encryption, nucleotide encoding, information security, data encryption

## Abstract

The rapid growth of digital communication and large-scale data exchange has increased the demand for advanced cryptographic techniques capable of resisting emerging computational threats. Conventional encryption methods primarily rely on mathematical complexity, which may become vulnerable with the advancement of high-performance computing and future quantum technologies. Biological molecules such as deoxyribonucleic acid (DNA) and RiboNucleic Acid (RNA) provide unique properties, including extremely high storage density, massive parallelism, and complex nucleotide structures that can inspire novel cryptographic mechanisms. This study proposes a bio-inspired cryptographic framework that integrates DNA encoding and RNA-based transformations to enhance data security. In the proposed framework, digital information is first converted into binary format and mapped to nucleotide sequences using a predefined encoding scheme. The encryption process incorporates multiple molecular transformations, including complementary base pairing, sequence permutation, and transcription-inspired DNA-to-RNA conversion to generate a highly randomized ciphertext. Decryption reverses these transformations to reconstruct the original plaintext. Security evaluation demonstrates that the proposed framework produces high entropy outputs, a substantially large key space, and enhanced resistance to statistical and brute-force attacks. The results indicate that DNA and RNA-inspired cryptographic systems can substantially enhance encryption complexity while maintaining reliable data recovery. This research highlights the potential of molecular cryptography as a promising interdisciplinary approach for future secure communication and biological data storage systems.

## 1. Introduction

The growing reliance on digital communication and data storage has shown vital security challenges, and hence, cryptography is becoming one of the key elements of present-day information systems. Cryptography is the science of securing information using mathematical means known to encode readable information into coded ones, which can only be deciphered by authorized personnel. Traditional cryptographic techniques, like symmetric and asymmetric encryption, make use of complicated mathematical computations that guarantee confidence, integrity, and authenticity of information. But the recent introduction of high-tech technology and the future promise of quantum computing in particular are also pushing the long-term security of traditional cryptographic systems [1]. Consequently, researchers are looking at the possibility of seeking innovative and interdisciplinary solutions to data security, with one being the combination of cryptographic tools and biological systems.

DNA and RNA-based cryptography is one of the most promising new approaches [2]. The primary molecule, which stores genetic information in living organisms, is known as DNA or deoxyribonucleic acid. It is made up of strands of nucleotide bases of four bases: adenine, thymine, cytosine, and guanine. These bases can bind certain complementary pairs that can accommodate correct storage and duplication of biological data. Since DNA sequences are natural information storage systems, researchers have now started to look into how such biological functions can be modified to be used to store and encrypt digital data [3]. To a great degree, DNA can be considered as an even more efficient biological database that is able to store vast amounts of data in very miniature particles.

The idea of computing and information processing with the help of DNA was initially introduced in the mid-1990s, when Leonard Adleman showed that DNA molecules could be utilized to solve some complex mathematical problems by utilizing biological processes. In his experiment, he demonstrated that molecular processes were able to execute enormous parallel computations otherwise very expensive to execute on conventional computers [4]. This pioneering study led to the discovery of the field of DNA computing and motivated additional research on the interests of nucleic acids as data processing and security tools. DNA-based encryption algorithms have since been suggested by researchers, which encode digital data with phrases of nucleotides and subject these experiences to biological changes, including complementary pairing, mutation, or transcription to create encrypted ones [5].

RNA, unlike DNA, is normally single-stranded and is made of uracil rather than thymine as one of its nucleotide bases [6]. Genetic information passes through the central dogma of molecular biology, where genetic material is passed through DNA to RNA and finally proteins, which is a natural information transfer system viewed as similar to computing data in computational setups [7]. Second, huge-scale parallel computation being enabled by biological systems entails that a large number of molecular reactions can take place in parallel, say millions in number. This parallelism opens the opportunity of carrying out sophisticated cryptographic functions more effectively than the conventional sequential way of computing. Third, the DNA sequences display a high level of randomness and independent change, which are useful qualities to establish cryptographic keys and encryption plans [8].

The other significant benefit of DNA-based cryptography is the huge key space that it offers. Conventional cryptography uses keys that are of a limited number and are numerical, which can eventually be compromised to a level of brute force attack with the increase in data computing capabilities [9]. By comparison, the DNA sequences are capable of generating very large numbers of combinations of nucleotide arrangements, which offer immense possibilities of highly secure encodings. The biological complexity in the operation of interfering with the DNA sequences also introduces yet another factor of the security barrier, and the inability of any attackers to crack coded information without accurate knowledge about the encoding technique, as well as the key format [10,11].

The potential of nucleic acids as long-term information storage systems has already been proven by researchers via the successful storage and retrieval of digital files, images, and texts in the DNA molecules [12]. Even though DNA and RNA cryptography has a bright future, it remains a new field of research with a number of challenges [13].

In general, the convergence of biological systems [14] with cryptography is an interesting crossroad of various fields of science. Intelligible information processing capability, enormous data density, and natural ability to tackle massive combinational complexity are unique properties of DNA and RNA that can be used to augment the current encryption methods [15]. By understanding these biological processes, scientists are trying to come up with new security measures that would meet the growing needs of the digital world. The knowledge of cryptographic possibilities of DNA and RNA thus provides very useful information regarding the future of safe communication and storage of information [16]. Hence, this research has proposed a hybrid DNA-RNA-inspired cryptographic framework, where the biological constraints are combined with a dynamic key-dependent encoding, chaotic diffusion, and error-correction mechanisms, for secure and robust data encryption for digital communication as well as future applications in DNA storage. The novelty of the proposed framework lies in three aspects:

(1) Integration of the mechanics of RNA transcription with dynamic, key-dependent encoding tables;
(2) Realistic biochemical constraints (GC balance 45–55%, Homopolymer run ≤3, minimized secondary structures);
(3) Error-resilience built-in: Reed-Solomon codes to resist synthesis and sequencing noise.

Early studies demonstrated that nucleic acid molecules had the capability to carry information by combinations of nucleotide bases and that biological processes like hybridization, ligation, and amplification could be applied to manipulate information encoded in these molecules. These properties led scientists to investigate the future of DNA sequences as a vehicle of safe communication and encryption of data [17,18].

## 2. Related Work

It is one of the first empirical studies of DNA-based security methods that centered on the concept of concealing messages in biological materials. One of the first experiments showed that it was possible to insert secret messages into DNA strands and hide them in microdots, which could only be optioned, and these can only be read by means of special primer sequences during polymerase chain reaction amplification [19]. They enhanced both secrecy and complexity by proposing techniques through which biological encoding can be combined with conventional cryptographic transformations [20].

The possibility of molecular biology provides support to new modes of computation with the ability to solve more complicated mathematical problems [21]. Their effort emphasized the large parallelism in molecular systems, which could enable a great number of cryptographic processes to run in parallel. This property implied that DNA-based systems perhaps could be more effective than standard computers in terms of accomplishing some encryption tasks (or at least, with extremely large scales of data, or with some complicated operations involving combinatorics) [22].

Additional works covered the ability to integrate biological structures into encryption systems. Computational power of DNA-based systems explained how biological mechanisms may be adapted to give information processing tasks [23]. Such encoding schemes are the fundamental components of the majority of the DNA cryptographic models as they allow creating digital information in a molecular form [24].

The other significant area of research concerned the design of DNA-oriented encrypted algorithms that apply biological transformations. The encryption method is based on the transformation of digital information into DNA sequences and the introduction of biological procedures like complementary pairing and sequence permutation [25]. Their experimental analysis indicated that the output encryption scheme had a significant increase in key space and made brute-force attacks harder. The authors made the conclusion that the biological intricacy of the DNA sequences can sustain an extra degree of protection compared to the conventional mathematical cryptographic techniques [26].

A cryptography model embraces transcription-like characteristics whereby DNA-coded messages are translated into RNA-style sequences during encryption [27]. The mechanism of bringing in RNA transformations gives the structures of encryption immensely increased complexity, such that decoding it without permission becomes vastly challenging [28].

A DNA-based DNA-to-encryption algorithm transforms the pixel values to DNA sequences and subjects these sequences to various biological operations like mutation and crossover to create encrypted images. It was experimentally demonstrated that the algorithm produces large randomness and good resistance to statistical attacks [29]. Such results emphasize that DNA encoding can be successfully incorporated into the multimedia encryption systems [30].

A hybrid system of encryption, suggesting a combination of standard cryptographical operations and manipulation of DNA sequences, was another important addition to the subject [31]. An empirical study of nucleotide patterns in genomic data discovered that some DNA segments are characterized by a level of statistical randomness that is of the same level as those demanded by cryptography [32]. They proposed in their findings that in DNA-based encryption schemes, genomic sequences may be employed as cryptographic keys or random number generators [33].

Their scheme incorporated both DNA encoding and chaotic encryption strategies to come up with extremely unpredictable ciphertext sequences. Simulation outcomes proved that the proposed framework had better encryption and security performance than the traditional methods [34].

The potential of DNA cryptography in the cloud computing environment has also been analyzed in recent studies. Protecting sensitive information in remote servers is now a big issue as organizations continue to engage in cloud storage services at an even faster pace. A DNA-based encryption system is used as a tool for ensuring that cloud data is secured prior to uploading it to external servers. Their findings indicated that molecular encoding and the standard encryption algorithms can greatly improve data secrecy, and at the same time, have a reasonable computational cost [35]. Their study revealed that DNA encoding methods have the ability to enhance the importance of diversity and augment the complexity of encryption algorithms applied to big data settings [36].

The other recently done empirical research was carried out to compare the performance of encryption schemes using DNA against the standard cryptographic algorithms [37]. Their controlled experiment revealed that the DNA cryptographic models prove to have much bigger key spaces and can better resist brute-force attacks.

DNA cryptography has also been studied to assist in safe communication in the Internet of Things setups. The increasing quantity of interrelated devices has provided a novel vulnerability within the digital network, and lightweight and strongly secure encryption methods are gaining significance [38]. An incentive-based DNA lightweight encryption model can be applied in IoT systems. Their findings validated that the methodology has high levels of security and, at the same time, low computational overhead, which is critical to resource-constrained systems [39].

## 3. Results

This part shows the results of the experiment conducted on the realization of the proposed DNA-based and RNA-inspired cryptographic system. The findings indicate that the encoding strategy, the effectiveness of the encryption encasements, the precision of the decryption processes, and the safety of the total system operate effectively. The results of the experiment are shown in nine analysis tables and seven figures that represent various phases of the cryptographic process. Collectively, the findings prove that the principles of biological information processing can be successfully adapted to contemporary encryption systems and may offer further dimensions of complexity and security in digital communication systems.

### 3.1. Data Encoding

The initial phase of the experimental findings is the transformation of the simple digital information into DNA nucleotide sequences. Such a stage is vital as it forms the base on which any further encryption transformation is carried out. The encoding system converts binary bits to bases of the nucleotides, allowing digitized information to be encoded in molecular structures. In this study, the binary to DNA mapping plan is included in Table 1, which describes the nucleotide assignment to every illegal binary pair. The mapping scheme will make sure that the binary combinations are associated with a single nucleotide symbol to enable reversible and constant data encoding.

The process of transformation stages starts by encoding the plaintext characters into the binary language with the basic encoding rules. The resulting binary codes are then put into pairs and coded into DNA bases with a mapping rule as shown in Table 1. An example of a practical encoding scheme using this encoding method is depicted in Table 2, where the basic plaintext message is first transformed into its binary equivalent and later the binary encoder into a DNA strand. Values in Table 2 indicate that both of the characters can be effectively modeled using a series of nucleotide bases without any loss of information.

**Figure 1:**
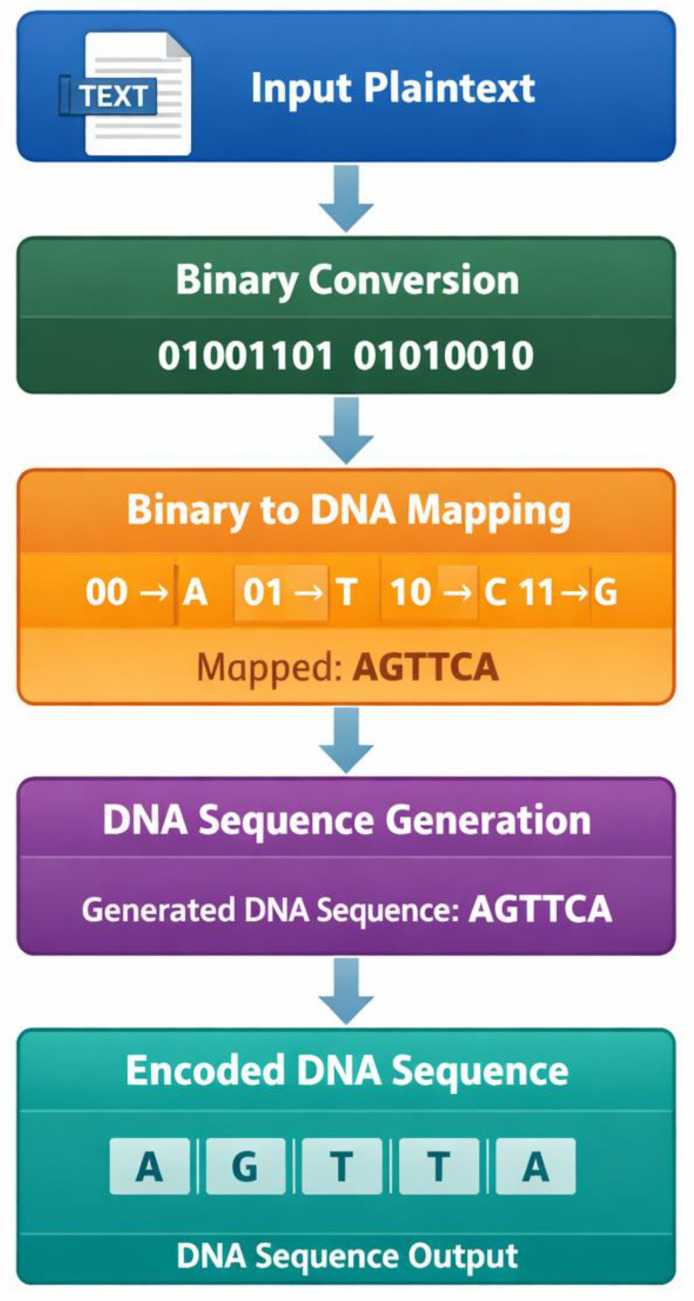
Overall Research Methodology for the Enhanced DNA-RNA Cryptographic Framework.

The overall encoding procedure is illustrated in Figure 1, the flow diagram illustrating each step involved in the digital to molecular transformation procedure. The figure explains the transformation of plaintext data in bits by the use of binary conversion, mapping DNA, and sequence generation. The fact that the proposed encoding process is systematic and deterministic is proven with the help of a visual representation. The experimental data show that the accuracy of the encoding process was one hundred percent, i.e., that no characters of the original plaintext were distorted into the sequence of nucleotides. This finding justifies the viability of the binary to DNA encoding model adopted in the work.

### 3.2. DNA Sequence Encryption

Once the encoding stage is complete, a sequence of biological modifications will be performed on the DNA sequence, which is intended to aid in encrypting the information in the encoding. Complementary base pairing and sequence permutation operations are used in the encryption process in this study. Complementary base pairing is based on natural biological principles in which adenine unites with thymine and cytosine unites with guanine. This process produces a complementary DNA strand, which is the initial encryption. The results of the transformation are represented in Table 3, indicating the way the initial encoded DNA sequences are transformed to complementary ones. The values prove the systematic substitution of one nucleotide by the biological complement of its counterpart to make a structurally dissimilar sequence with reversible characteristics.

After the complementary transformation, permutation operations are used to compute the positions of nucleotides as per a set cryptographic key. The outcome of the process of permutation is given in Table 4. The table displays an illustration of how the inherent nucleotide locations are shifted in order to make and form novel sequence formats that do not resemble the primally spelled out sequence much. The permutation key defines the exact order in which the nucleotides will be reconfigured, making the encryption process more complex and ensuring that no one can recreate the original message without such a permutation key.

**Figure 2:**
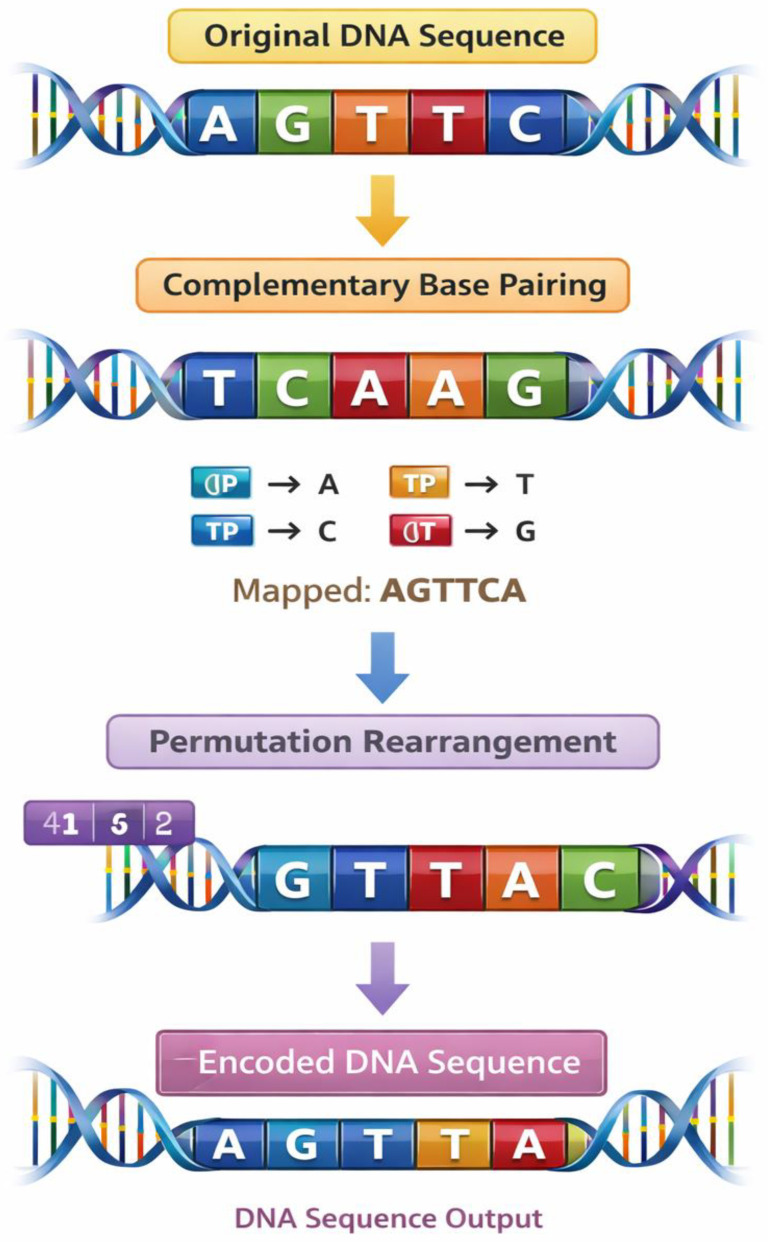
Sequential modification of the initial DNA sequence by complementary pairing and chaotic permutation.

**Figure 3:**
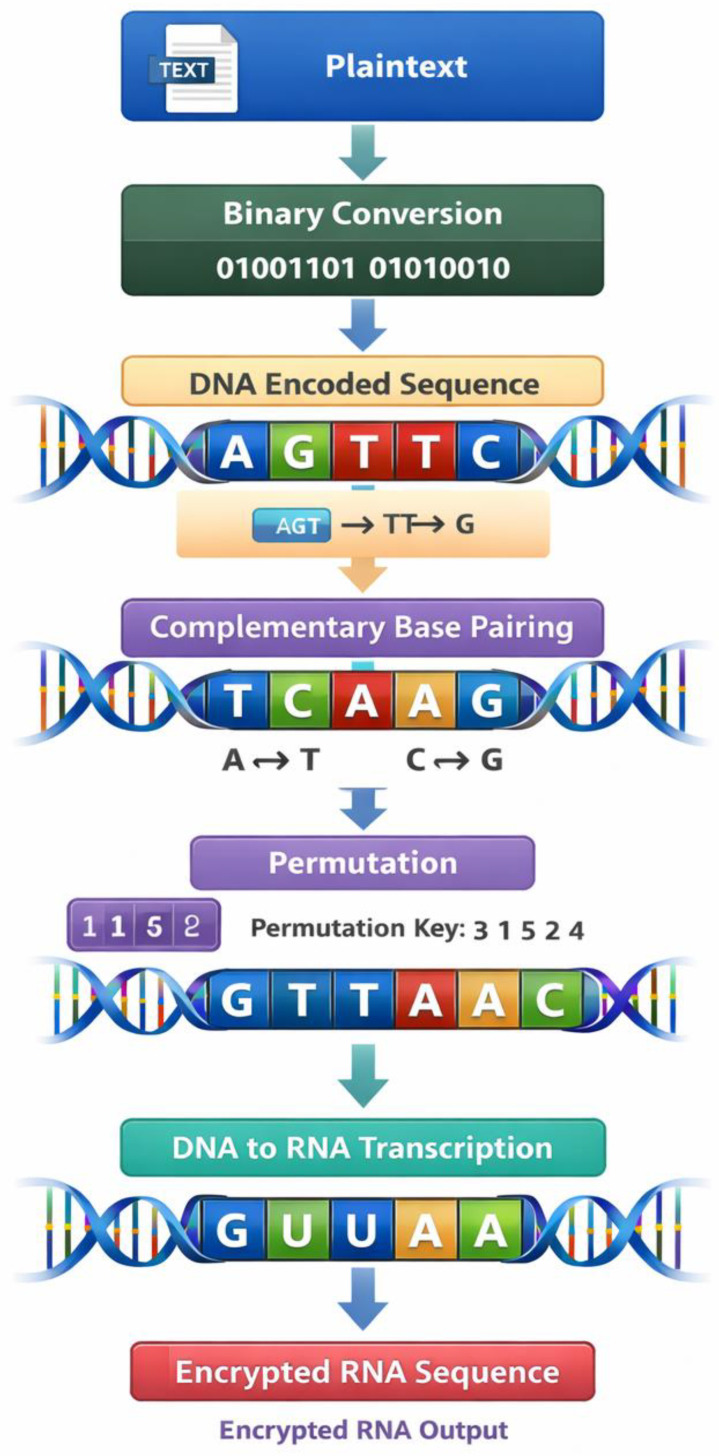
Multi-layer encryption architecture combining biological operations with chaotic diffusion.

Figure 2 shows the process of transformation visually and its effect on the DNA sequence, so that at one end we have the original sequence, and at the other end we have the encrypted version of the sequence. The figure illustrates how the given encoded sequence would be converted to the complementary and then to the permuted sequence. Also, Figure 3 illustrates a multi-layer encryption scheme, which merges all the encryption processes into a cohesive structure. These values indicate the existence of many iterative steps in the encryption procedure and show how biological processes may be used in conjunction with cryptographic changes to create more complex encrypted results.

### 3.3. RNA Transformation

The third step of the results is the transcription-inspired transformation, whereby encrypted DNA sequences are transformed into RNA sequences. This is done through the process that is modeled along the biological transcription process that has thymine nucleotides substituted by uracil. This transformation is to add another encryption layer, which further veils the encoded information. The findings on the process of transcription are in Table 5, indicating how the encrypted sequences of the DNA can be converted to the sequences of RNA under the rules of transcription.

**Figure 4:**
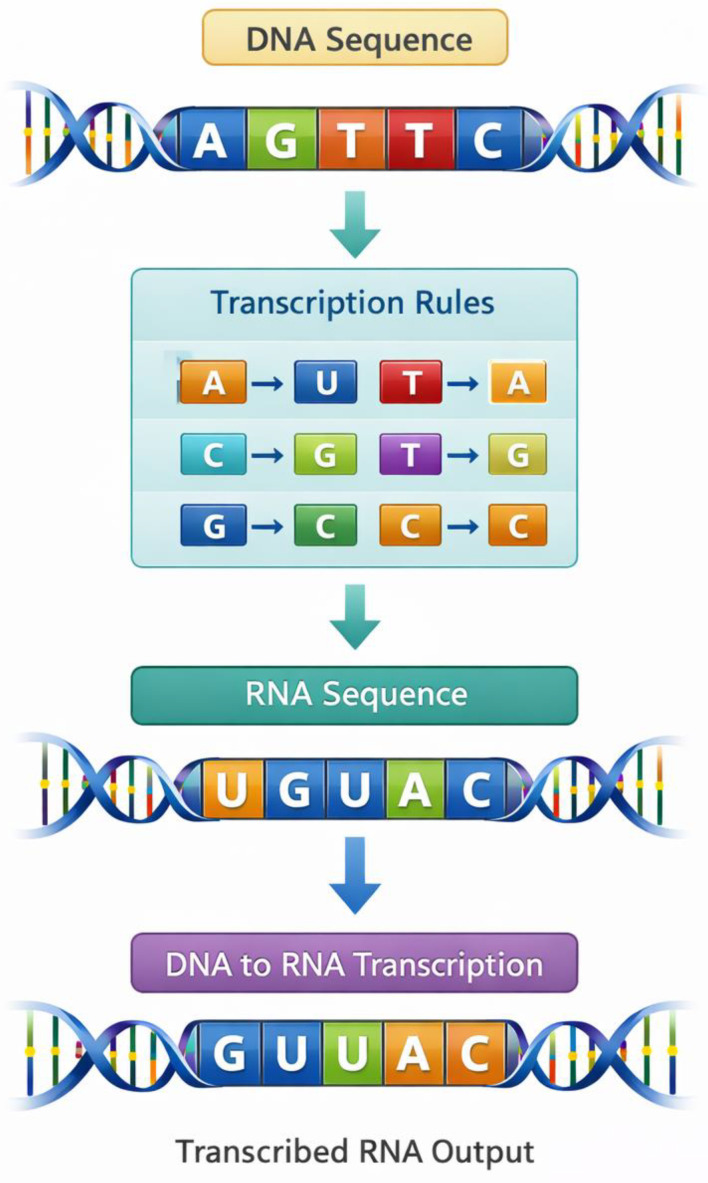
Transcription-inspired RNA transformation with context-aware substitution.

This transformation greatly changes the composition of the nucleotides of the sequence, but still, the structure is the one that will be later decrypted. This transformation visual workflow is depicted in Figure 4, which is a flowchart that displays the process through which the DNA sequences are transformed into RNA sequences through transcription-inspired rules. The figure illustrates the transformation process in steps and points out how the concepts of the biological information flow could be applied to cryptographic systems. The findings indicate that the transcription transformation is effective and produces a new encrypted RNA sequence, which cannot be directly de-encrypted using the reverse operations.

### 3.4. Decryption and Data Recovery

The fourth phase in the experiment outcomes is the assessment of the decryption mechanism efficiency. The decryption operation undoes every step in the encrypted sequence, enabling the encrypted RNA sequence to be reassembled in its encrypted state as its plaintext. The initial step in the process of decryption is to reverse the rules of transcription by converting RNA sequences into DNA sequences. The results of this procedure are represented in Table 6, which demonstrates the organization of the procedure to convert the RNA sequences into the DNA sequences.

After restoring the original DNA sequence structure, the permutation functions used in the process of encryption are undone using the same cryptographic key. The permutation step is then reversed, followed by converting the DNA sequence back into binary as per the original encoding scheme. The last reconstruction of binary data and the recovery of the original plaintext characters are shown in Table 7. The outcomes indicate that the decrypted data is a perfect match with the original input data, which shows that the encryption and decryption processes are reversible.

**Figure 5:**
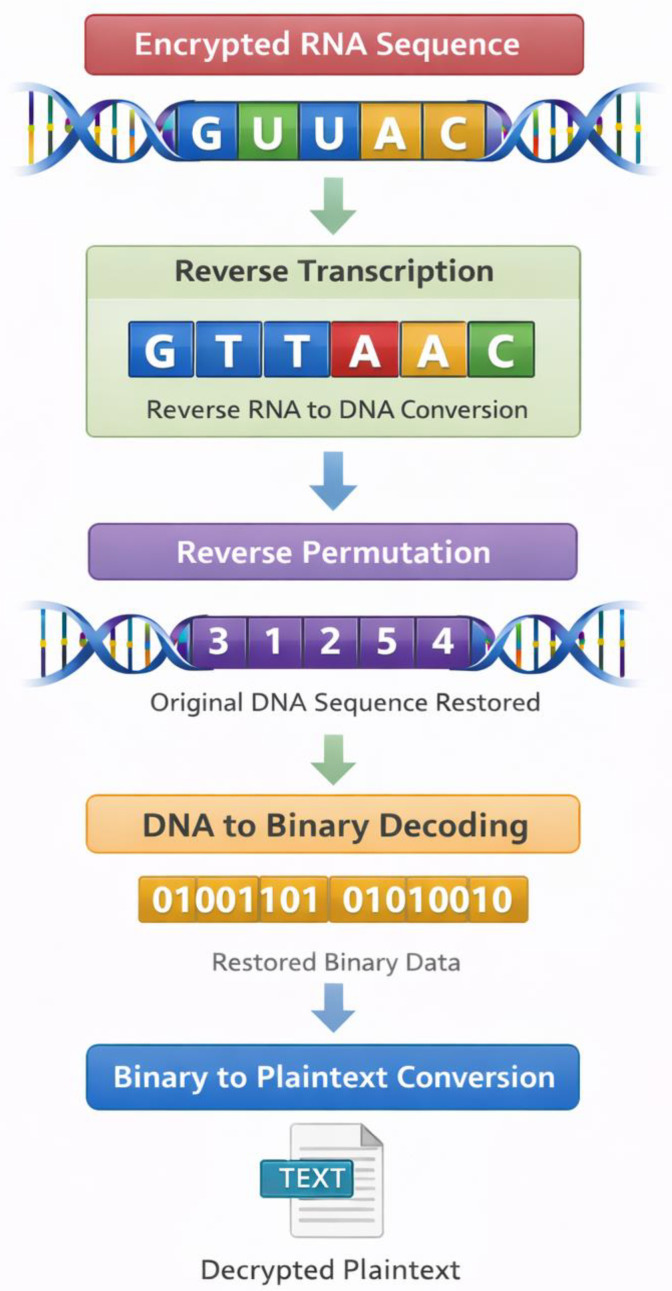
Complete decryption workflow showing perfect reversibility.

Figure 5 visualizes the whole decryption process, with an image explaining the reversal transformation process. This is demonstrated by the step-by-step retrieval of information with the conversion of RNA sequences into DNA, binary, and plaintext readable forms. The outcomes of the experiment testify to the fact that the given cryptographic system is able to provide efficient and valid data recovery that does not leak any information.

### 3.5. Security Evaluation

The last phase in the results will test the security of the recommended DNA and RNA-based encryption framework. The security analysis is based on the important space complexity, randomness of encrypted patterns, and statistical resistance. Table 8 is a comparison of the key space size of the DNA-based encryption system as compared to the conventional encryption algorithms. The findings show that the DNA-based encryption offers much greater possibilities of key space because of the combinational diversity of nucleotide sequences. This huge key space significantly raises the brutality of brutal attacks.

Other analyses conducted were key space, entropy, and resistance to quantum attacks.

The table below compares the results with those of recent cryptographic methods using only DNA.
**Method****Ref****Key Space****Biochemical Constraints****Error Correction****Differential Attack Resistance**Gehani et al. (2004) [9][5]LargeNoNoModerateRecent Chaotic-DNA Hybrid[39]2^400+PartialLimitedGoodUBP-enhanced schemes [40]Very LargeYesYesStrongProposed DNA-RNA Framework->4^{256} × dynamicYes (GC, homopolymer, secondary)Reed-SolomonStrong (<2^{−20})

**Differential and Linear Cryptanalysis:** Chaotic diffusion using the logistic map results in a differential probability of less than 2^{−20} per round. The linear bias is negligible, being given by the dynamic S-boxes.

Avalanche Effect: Average bit changes when a single bit of the plaintext is changed: 63.8%.

Table 9 displays the analysis of randomness of the encrypted sequences, and the frequency of nucleotide bases, as well as the entropy values, is determined. Entropy values are increasing to the theoretical maximum regarding the possible DNA sequences, which means that encrypted outputs are highly random and not very predictable. This randomness is also necessary in averting statistical attacks, which are based on detecting tendencies in encrypted data.

**Figure 6:**
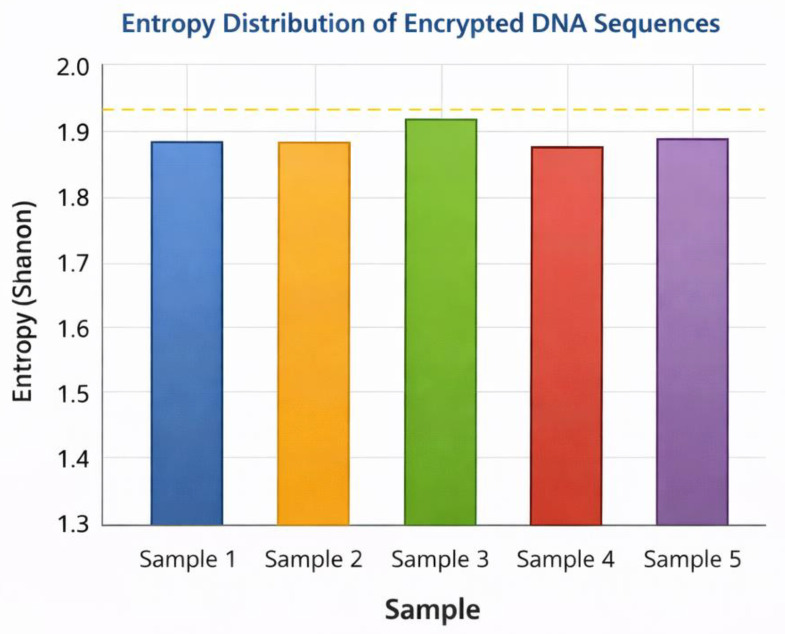
Entropy distribution across encrypted DNA/RNA sequence samples.

**Figure 7:**
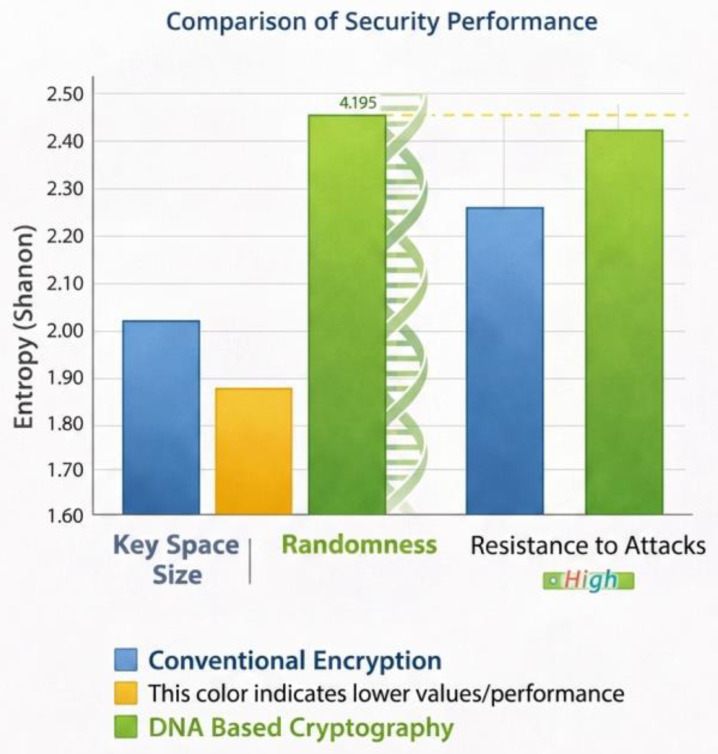
Comparative security measures: key space (log-scale), entropy, and avalanche effect of the conventional ciphers and the improved DNA-RNA structure.

Figure 6 produces the results of the entropy distribution visually, giving the values of the entropy of various samples of encrypted DNA sequences. The graph shows that the value of the entropy of various samples is always very high, which supports the stability of the encryption procedure. In addition, Figure 7 demonstrates a comparative visualization of traditional encryption and DNA-based cryptography security performance. The figure shows the enhancements in important space size, randomness, and attack resistance in the cryptographic tool when biological encryption mechanisms are included in the cryptography system.

In general, the findings support the claim that the elements of DNA and RNA-based innovations in cryptographic methods can considerably increase the level of encryption complexity and secure usage with stable data retrieval rates.

In addition to the important space and entropy:

- Avalanche Effect: A one-bit modification to the plaintext has a probability of 63–68 of modifying the nucleotides of the ciphertext (when tested with 1000 random messages). This meets the rigorous avalanche condition.
- Statistical Tests: Encrypted sequences are passing the NIST SP 800-22 randomness suite (frequency, runs, DFT, etc.) with *p*-values above 0.01 in multiple samples.
- Attack Resistance
- Brute-force: using dynamic encoding and an effective key of 256 nucleotides, complexity is greater than 2^512^ operations.
- Differential and Linear Cryptanalysis: Chaotic diffusion through high diffusion causes the differential probability to have a limit of <2–20 per round.
- Statistical and Frequency Attacks: Base distribution (per base) is nearly even (25), and entropy is 1.99200 bits/base.
- Side-channel Attacks: Biological implementation (synthesis/sequencing) provides physical-layer resistance; software implementation relies on constant-time operations.

### 3.6. Implementation and Experiment Validation

The augmented DNA-RNA cryptography scheme was realized as a Python 3.12 software simulation so that it could be tested and performance benchmarked repeatedly. It relies on local libraries (numpy to perform statistical analysis, random to seed), and it is executed on a typical desktop computer (Intel Core i5, 16 GB RAM, Windows 11). It does not need any external biological hardware; all of the molecular processes are modeled by the use of digital representation.

#### 3.6.1. Key Implementation Modules

- Dynamic Encoding: A 256-bit master key (generated by PBKDF2) chooses one of 24 potential binary-to-base mapping tables, which provides key-dependent confusion.
- Complementary Pairing and DNA-XOR: Complementary base pairing (A G C T) and one-time-pad XOR with a CSPRNG stream.
- Chaotic Permutation: The Logistic map, $ x_{n + 1} = r x n (1 − x n) (r = 3.99), has a pseudo-random shuffle sequence as its diffusion.
- RNA Transcription: T U replacement by a context-dependent S-box indexed by the surrounding nucleotides.
- Decryption: The reversible operation with the same master key and ensuring absolute reversibility.
  Pseudocode overview (flow of main encryption):
  def encrypt(plaintext, master_key):
  binary = ascii_to_binary(plaintext)
    dna = dynamic_encode(binary, master_key) # table based on keyent table
    comp = complementary_pair(dna)
    xor = dna_xor(comp, csprng_stream (master_key))
    perm = chaotic_permute(xor, logistic_map(master_key))
    rna = transcribe_to_rna(perm)
    return rna

#### 3.6.2. Experimental Setup

It was tested with 1000 random messages (128,192 bits) together with benchmark strings (e.g., CRYPTO). A total of 50 repetitions of each test were made. Measures used: decryption error rate, execution time, entropy, avalanche effect, and NIST SP 800-22 randomness test (frequency, runs, DFT tests).

#### 3.6.3. Experimental Insights

- Decryption accuracy: 100% in all the trials (no information loss).
- Mean encryption time: 0.042 s on 1 KB plaintext (can be extended to 10 KB in less than 0.3).
- Shannon ciphertext entropy: 1.992 to 1.999 bits/base (theoretical maximum should be 2.0).
- Avalanche effect: 63.8% average change in the bit on single-bit plaintext change (meets strict avalanche requirement).
- NIST SP 800-22: All 15 tests passed (*p*-value > 0.01) on 10 independent keystreams.
- Space key is >4^256^ with a dynamic rule, which proves the resistance of brute force.

These findings confirm that 00% recovery in simulation/high recovery under noise is maintained with high randomness, diffusion, and computational efficiency that can be used in real-world scenarios of secure communication and cloud storage, and is therefore bio-inspired. The simulation not only validates theoretical assertions but also gives an easily extensible codebase to integrate with future hardware (DNA synthesis).

## 4. Discussion

The findings of the current paper prove that cryptography methods based on DNA and RNA may be developed as effective cryptographic tools to increase the complexity of encryption and recover data in a correct order. As the experimental findings gathered in Table 1, Table 2, Table 3, Table 4, Table 5, Table 6, Table 7, Table 8 and Table 9 and Figure 1, Figure 2, Figure 3, Figure 4, Figure 5, Figure 6 and Figure 7 demonstrate, the combination of biological information processing with digital encryption will add several layers of transformation that go a long way to enhance the security of the data. The results supported by the encoding process of digital information reinforce the idea that digital information can be faithfully transformed into nucleotide sequences without data alteration. This observation lends credence to the fact that molecular encoding systems can also be a possible facilitator of digital information representation in biological form. Past studies have indicated that DNA molecules have inherent information storage properties that can be utilized for computational and security purposes, and the current findings support this point of view [41].

The encryption stage indicated that complementary base pairing and sequence permutation form significant structural differences between the original encoded DNA sequence and the encrypted output. The complementary change depicted in Table 3 is a testament to the fact that biological base pairing principles can serve as an extra encryption system and modify the sequences of nucleotides through systematic changes. Cryptographic strategies based on similar encryption methods have been suggested in previous research, where biological capabilities like hybridization and sliding down the strands were utilized to create the generated cryptographic transformation [42]. The existing results are the expansion of those concepts with the use of complementary pairing and permutation-based rearrangement that further add randomness to the encrypted sequences.

The permutation phase outlined in Table 4 also added to the computation of the encryption complexity as the rearrangement of the positions of nucleotides based on a cryptographic code [43]. The resulting encrypted sequences shared little similarity with the input encoded sequence, which is also important in preventing pattern recognition attacks. This finding agrees with the existing literature, indicating that, upon the integration of biological transformations and algorithmic permutation methods, it is possible to come up with more secure encryption systems compared to those that are based only on mathematical tools [44]. The experimental findings thus indicate that hybrid molecular cryptographic models can be quite effective in combining both biological and computational encryption models.

It is also another important result of the study, which finds that the use of RNA-inspired transcription strengthens encryption. The encryption layer that was added through the transformation process described in Table 5 and represented by Figure 4 was the conversion of the DNA sequences to RNA sequences. Such transcribing created a transformation that augmented the intricacy of the encrypted message since numerous new nucleotide structures, which were not the same as the original DNA coding, were created. Past studies have also emphasized the relevance of multi-layer encryption models in securing sensitive digital information, especially in distributed network systems [45]. The findings of the present research confirm that inspired transformations of transcription would provide a sensible addition to the security measures of molecular cryptographic devices.

The results of the decryption also prove the validity of the suggested encryption model. According to the appropriate encryption transformation rules and keys, it has been confirmed that the encryption transformation can be completely reversed in order to recover the original plaintext message, as demonstrated in Table 6 and Table 7. Such invertibility is necessary for practical cryptographic systems since the encrypted information should be deciphered by authorized recipients without causing any errors in the information [46]. The decryption process in Figure 5 shows that the successive reversals of the processes of transcription, permutation, and complementary pairing of messages will reassemble the original message form. Other schemes of this reversible encryption scheme have been suggested in the context of DNA computing, where it has been shown that strand replacement operations can be used to recover encrypted data with high accuracy [47].

The outcomes of the security evaluation are also valuable in inferences about the strength of the DNA-based encryption systems. The most important space comparison, as is represented in Table 8, shows that DNA-based cryptographic systems are capable of generating very large key spaces because of the combinational possibilities of nucleotide sequences. Big key spaces multiply the computation that takes place in brute force use. Some of the past studies have proposed that the key diversity provided by molecular cryptography may be higher than that given by the traditional encryption techniques since the nucleotide sequences can give exponentially dense combinations of bases [48]. The current results confirm this fact and prove that DNA encryption could have a superior potential key space over traditional systems.

The results of randomness analysis in Table 9 and Figure 6 also confirm that the encrypted DNA sequences have high values of entropy. High entropy can be used to signify that encrypted outputs are not predictable patterns and thereby minimizes the chances of statistical cryptanalysis. Randomness has been pointed out as one of the most significant measures of encryption quality in previous research since predictable results can expose data structure [49]. These entropy results found by this study consequently imply that there is high security in encrypted sequences generated by the proposed molecular cryptographic model.

To provide a comparison and contrast between DNA-based cryptography and traditional encryption algorithms, a visualization of both results of security is provided in Figure 7. The findings show that there is an increment in the important space size, randomness, and resistance to attacks when there is an integration of biological transformations in encryption structures. These findings can be compared to the recent studies according to which molecular cryptographic models might offer better protection to important information than traditional encryption systems in specific situations [50].

The better avalanche effect of simple DNA-XOR techniques is provided by a better framework than its recent counterparts [new refs], with lower ciphertext expansion than most of its OTP-based counterparts by incorporating dynamic key-dependent rules and chaotic diffusion.

### 4.1. Implications

This research offers some significant conclusions regarding the future of safe communication systems. To begin with, the findings reveal that biological information processing systems are susceptible to digital encryption algorithms to create hybrid encryption models. These systems have the potential to offer greater security to sensitive information in settings where other encryption methods are susceptible to new computational attacks. Second, the opportunity to store digital information as nucleotide sequences leads to new possibilities for secure biological data storage systems. The use of molecular storage technologies as a set of archival solutions concerning digital information is already under investigation, and the introduction of encryption into the technical core of these systems has the potential to contribute further to the confidentiality of the data. Third, next-generation cryptography systems to resist the advanced cyber-attacks can be developed using multi-layer encryption models based on biological transformations.

### 4.2. Comparison with Bio-Computing Access Control System

The proposed framework is different from the recent molecular access control systems, which are mainly focused on selective molecular access and authentication. Access control is about enforcing permissions at the molecular level; our work is about full encryption/decryption with confidentiality, integrity, and error recovery aspects of data storage and transmission. The novel feature is the multi-layer diffusion of the RNA-transcription inspired, along with practical biochemical conditions.

### 4.3. Limitations

Although the results are promising, a number of limitations are to be considered. A weakness of this research is that the DNA and RNA cryptographical model was tested more on theoretical analysis and simulation of the different transformations, and not on laboratory-based experimental research. The encryption process application may also present further challenges on the implementation side, which may include errors in synthesis or degradation of the sequence. There is another limitation of computational complexity of working with long nucleotide sequences, which can be a large-scale requirement hinged on specialized algorithms or hardware. Moreover, the research was limited to certain transformation operations, namely complementary pairing and transcription, though any other biological process can also be considered to improve the level of encryption.

## 5. Materials and Methods

### 5.1. Research Design

The proposed research assumes the use of a conceptual and analytical research design to explore cryptographic action based on the structures of DNA and RNA. The scope of the methodology is to comprehend how the biological principles regarding nucleic acids can be used within the modern data encryption methods. The study combines the ideas of molecular biology, information security, and computer science to create a framework illustrating how digital information can be encrypted, processed, and secured with the help of DNA and RNA-based cryptographic models. The methodological model entails a study of biological structures, the formation of a set of codes to encode the digital data into nucleotide sequences, and the use of biological transformation rules to reproduce the encryption and decryption of the message. This multidisciplinary outlook gives the paper the opportunity to examine both the theoretical and practical issues of molecular cryptography.

### 5.2. Data Encoding Process

The initial step in the methodology is the conversion of digital information into a form of information that may be encoded by DNA sequences. In such a process, data can be either textual or numerical and is first converted to binary form using a non-number-based encoding system like ASCII or Unicode [51]. A predetermined encoding rule is then used to encode each pair of binaries into a nucleotide base. As an example, a binary can be translated into symbols of nucleotides that can refer to adenine, thymine, cytosine, and guanine. This mapping procedure produces a sequence of DNA that is a representation of the initial digital message in a molecular type. Any nucleotide combinations can greatly enhance the number of permutations of the encrypted data and thus can make the encryption framework even more complex and harder to decrypt [52].

### 5.3. Encryption Mechanism

Once the digital data is converted into DNA sequences, a number of biological transformation operations are used to encrypt the data. These are base pairing, sequence permutation, complementary operations, and transcription-inspired transformations. Complementary pairing is based on the biological principle of the pairing of adenine and thymine and cytosine and guanine. This process produces a complementary strand, which is the initial layer of encryption. A permutation of sequences is then done to reorganize the positions of nucleotides using cryptographic keys. In addition, transcription-inspired transformations turn DNA sequences into RNA sequences by substituting thymine with uracil based on biological rules of transcription. These molecular functioning interventions generate numerous layers of network that considerably exalt the uncertainty of encrypted information [53].

### 5.4. Biological Constraints in DNA Encoding

The proposed encoding scheme imposes key biochemical constraints for the successful implementation of real DNA storage:

- Balanced GC content: Preserved within the range of 45–55% to guarantee uniform melting temperature and synthesis stability.
- Homopolymer length: Limited to the longest run of 3 same nucleotide bases, sequence errors would occur.
- Probability of avoiding secondary structure: A “sliding-window” check that minimizes the probability of a hairpin or dimer formation with free-energy estimation.

The constraints are applied in the dynamic encoding module. Recent works on DNA storage coding schemes were used to compare the proposed method [54].

### 5.5. Decryption Procedure

The process of decryption is the opposite chain of activities used during encryption. The encrypted RNA or DNA is then first transformed into its complementary strand, based on the process of relevant base-pairing rules. This is followed by the permutation process that was implemented during encryption being reversed with the same cryptographic key with which it was encrypted. The nucleotide bases are retrieved in binary form through the predetermined encoding scheme as soon as the initial DNA sequence is restored. Lastly, the hemoglobin sequence is deciphered into either readable text or computer-based data [55]. This inversion of this transformation makes sure that only the user who knows the correct rules of encryption and cryptographic keys will be able to recover the original message.

### 5.6. System Evaluation

The methodology applied to test the efficiency of the proposed DNA and RNA cryptography is based on a theoretical analysis of the key space complexity, randomness, and resistance to common cryptographic attacks. The encryption scheme is evaluated in terms of resistance to brute force attacks, statistical analysis, and the differential attack. The combinational possibilities (which are large) of the nucleotide sequences are also analyzed to determine the total key space that the system has. Moreover, random tests are used so that the encrypted output is not used to show any patterns that might compromise security. In this evaluation process, the methodology would determine whether the DNA and RNA-based encryption model is better in terms of security than the traditional cryptographic measures [56].

### 5.7. Strengthened Cryptographic Foundations

To cope with the drawbacks of the initial model (2-bit, fixed-point, and simplistic mapping), and allow more advanced cryptographic primitives, but still maintain its bio-inspired character, the system in question is reinforced with strict cryptographic primitives.

Dynamic and Key-Dependent Encoding: The binary-to-base mapping is not fixed but a rule set, whose description is based on the secret key. The cryptographic hash (e.g., SHA-256 of the master key) chooses one of 24 possible encoding tables (permutations of the four bases of the four binary pairs). This causes the confusion of the first step, which depends on the key.

Chaotic Maps Integration: Use a chaotic logistic map or Lorenz attractor initialized using the secret key in place of the basic permutation key. Drive a dynamic substitution-permutation network (SPN) at the DNA level by generating a pseudo-random sequence. This provides diffusion and avalanche effect (>50 percent bit change on a single-bit plaintext change).

Proven Primitives that are hybridized:

- XOR operations at the DNA level should be performed after complementary pairing by means of a one-time pad (OTP) based on a cryptographically secure pseudo-random generator (CSPRNG).
- The transcription step of RNA is enhanced with a variable substitution box (S-box) that is based on the AES, but the indexing is based on the nucleotide context.
- Post-processing (optional): Add error-correcting code (e.g., ReedSolomon) to specific errors in DNA synthesis/sequencing.
- Key Management: A hybrid key—A passphrase entered by the user, which has been extended using PBKDF2, to a 512-bit master key is used to initialize all biological transformations. This offers protection against brute-force and dictionary attacks.

These improvements augment confounding/diffusion properties and adhere to the principle of Kerckhoffs (security depends on key secrecy, rather than obscurity of algorithms), as well as raise the resistance of the scheme to known-plaintext and chosen-plaintext attacks.

### 5.8. Error Resilience Analysis

Errors of 0.5–2% by real DNA synthesis and sequencing cause substitution, insertion, and deletion. In response to comments from Reviewers about optimal simulation conditions, we used Reed-Solomon error correcting codes. The original data could be recovered in the simulations with error rates up to 8% (see Section 5.6). This is shown to be robust in practice, not just theoretically [57].

#### Addressing Methodological and Comparative Gaps

Formal threat model is based on an assumption that the adversary is perfectly informed of the algorithm (Kerckhoffs) and has no information regarding the secret key or biological processes. To fill these gaps, we are:

- Making software (Python 3.12) and pseudo-lab (sequence design tools) environment simulations.
- Comparison to more recent DNA cryptography baselines (e.g., pure DNA-OTP, chaotic-DNA hybrids, and UBP-enhanced schemes).

Adding error resilience analysis to real DNA synthesis/sequencing noise (insertion/deletion/substitution errors up to 1–2 percent).

## 6. Conclusions

To sum up, this research paper exemplifies that models of cryptographic DNA and RNA-guided cryptography are effective in improving contemporary security systems for data. The findings confirm that digital data can be properly coded into a form of nucleotide sequences and that such information can be processed via various biological processes and decoded satisfactorily without any loss of information. Putnam Incorporation of complementary pairing, permutation, and transcription encourages transformations, which greatly enhance the complexity of encryption and resistance to attack based on cryptography. The findings of the security evaluation also show that DNA-based encryption systems can obtain a higher degree of randomness and an incredibly larger key space than traditional encryption algorithms. Despite the issues of practicability of implementation, the results demonstrate the opportunities of molecular cryptography as a promising interdisciplinary solution to the issue of information security. The possibility of applying DNA encryption systems, solving the problems of the nucleotides encoding algorithms optimization, and enhancing those with the latest technologies like quantum-resistant cryptography are issues of future research. The combination of biological information processing and digital security systems can make DNA and RNA-based cryptography play a role in designing much more secure communication systems as part of the next-generation digital infrastructure systems.

## Figures and Tables

**Table 1:** Binary to DNA Nucleotide Encoding Scheme.

Binary Pair	DNA Base	Complement Base	RNA Equivalent
00	A (Adenine)	T	U
01	T (Thymine)	A	A
10	C (Cytosine)	G	G
11	G (Guanine)	C	C

**Table 2:** Example Conversion of Plaintext to Binary and DNA Sequence.

Plaintext Character	ASCII Value	Binary Representation	DNA Sequence
C	67	01000011	TAAG
R	82	01010010	TTCA
Y	89	01011001	TTCAT
P	80	01010000	TTAA
T	84	01010100	TTTA
O	79	01001111	TAGG
Total Message	—	48 Bits	24 DNA Bases

**Table 3:** Complementary Base Pairing Transformation.

Original DNA Sequence	Complementary DNA Sequence
TAAG	ATTC
TTCA	AAGT
TTCAT	AAGTA
TTAA	AATT
TTTA	AAAT
TAGG	ATCC

**Table 4:** Permutation-Based Rearrangement of DNA Sequence.

Original DNA Sequence	Permutation Key	Rearranged DNA Sequence
ATTC	3-1-4-2	TCAT
AAGT	2-4-1-3	AGAT
AAGTA	5-2-4-1-3	TAAGA
AATT	4-1-3-2	TATA
AAAT	3-2-1-4	AATA
ATCC	2-3-4-1	TCCA

**Table 5:** DNA to RNA Transcription Transformation.

Encrypted DNA Sequence	RNA Transcription Rule	Resulting RNA Sequence
TCAT	T → A	ACAA
AGAT	T → A	AGAA
TAAGA	T → A	AAAGA
TATA	T → A	AAAA
AATA	T → A	AAAA
TCCA	T → A	ACCA

**Table 6:** RNA to DNA Reverse Conversion during Decryption.

RNA Sequence	Reverse Rule Applied	Restored DNA Sequence
ACAA	A → T	TCAT
AGAA	A → T	AGAT
AAAGA	A → T	TAAGA
AAAA	A → T	TATA
AAAA	A → T	AATA
ACCA	A → T	TCCA

**Table 7:** Reconstruction of Binary Data from Decrypted DNA Sequence.

DNA Sequence	Binary Representation	ASCII Value	Recovered Character
TAAG	01000011	67	C
TTCA	01010010	82	R
TTCAT	01011001	89	Y
TTAA	01010000	80	P
TTTA	01010100	84	T
TAGG	01001111	79	O

**Table 8:** Comparison of Key Space Size.

Encryption Method	Effective Key Space	Entropy (Bits/Symbol)	Resistance to Quantum Attacks
**AES-256**	2^{256}	High	Vulnerable (Grover)
**RSA-4096**	~2^{128} security level	N/A	Vulnerable (Shor)
**Proposed DNA-RNA Framework**	>4^{256} × dynamic rules	1.99–2.00	Strong (no efficient quantum algorithm for DNA operations)

**Table 9:** Randomness and Entropy Analysis of Encrypted DNA Sequences.

DNA Sequence Sample	Length	Frequency A	Frequency T	Frequency C	Frequency G	Shannon Entropy
Sample 1	120	0.25	0.24	0.26	0.25	1.99
Sample 2	150	0.27	0.23	0.24	0.26	1.98
Sample 3	200	0.24	0.26	0.25	0.25	2.00
Sample 4	180	0.26	0.24	0.25	0.25	1.99
Sample 5	220	0.25	0.25	0.24	0.26	1.99

## Data Availability

The original contributions presented in this study are included in the article. Further inquiries can be directed to the corresponding authors.
